# Editorial Comment: Randomised Trial of Adjuvant Radiotherapy Following Radical Prostatectomy Versus Radical Prostatectomy Alone in Prostate Cancer Patients with Positive Margins or Extracapsular Extension

**DOI:** 10.1590/S1677-5538.IBJU.2020.05.10

**Published:** 2020-07-31

**Authors:** Felipe Lott

**Affiliations:** 1 Instituto Nacional do Câncer - INCA Rio de Janeiro RJ Brasil Instituto Nacional do Câncer - INCA, Rio de Janeiro, RJ, Brasil

Hackman G ^1^, Taari K ^2^, Tammela TL ^3^, Matikainen M ^2^, Kouri M ^4^, Joensuu T ^5^, et al.

## COMMENT

The latest guideline suggest adjuvant radiotherapy or observation following prostatectomy with adverse pathological findings (1, 2). This trial randomized 250 patients (1:1) with pT2N0M0 with positive margins or pT3aN0M0 and tries to answer if there is a benefit from adjuvant radiotherapy after radical prostatectomy. The postoperative PSA was less than 0.5. The median follow-up for alive patients was 9.3 yr and 8.6 yr in the adjuvant and in the observation group respectively. The primary endpoint was biochemical recurrence-free survival (BCRFs) and the overall survival, cancer-specific survival, and adverse events were the secondary ones.

The authors founded a 74% benefit of BCRFs in the adjuvant group, and the number needed to treat was 4. There were no difference in the overall survival and in cancer-specific survival, with more grade 1 and 2 adverse events in the adjuvant group.

The first results of the RADICALS trial (NCT 005541047) recently presented at ESMO Congress 2019 did not show any benefit in BCRFs (secondary endpoint), supporting the use of early salvage radiotherapy.
